# T1-mapping in the heart: accuracy and precision

**DOI:** 10.1186/1532-429X-16-2

**Published:** 2014-01-04

**Authors:** Peter Kellman, Michael S Hansen

**Affiliations:** 1 National Heart, Lung, and Blood Institute, National Institutes of Health, Bethesda, MD, USA

**Keywords:** T1 map, Accuracy, Precision, Reproducibility, ECV, Off-resonance, MOLLI, SASHA, ShMOLLI, Cardiovascular magnetic resonance

## Abstract

The longitudinal relaxation time constant (T1) of the myocardium is altered in various disease states due to increased water content or other changes to the local molecular environment. Changes in both native T1 and T1 following administration of gadolinium (Gd) based contrast agents are considered important biomarkers and multiple methods have been suggested for quantifying myocardial T1 in vivo. Characterization of the native T1 of myocardial tissue may be used to detect and assess various cardiomyopathies while measurement of T1 with extracellular Gd based contrast agents provides additional information about the extracellular volume (ECV) fraction. The latter is particularly valuable for more diffuse diseases that are more challenging to detect using conventional late gadolinium enhancement (LGE). Both T1 and ECV measures have been shown to have important prognostic significance.

T1-mapping has the potential to detect and quantify diffuse fibrosis at an early stage provided that the measurements have adequate reproducibility. Inversion recovery methods such as MOLLI have excellent precision and are highly reproducible when using tightly controlled protocols. The MOLLI method is widely available and is relatively mature. The accuracy of inversion recovery techniques is affected significantly by magnetization transfer (MT). Despite this, the estimate of apparent T1 using inversion recovery is a sensitive measure, which has been demonstrated to be a useful tool in characterizing tissue and discriminating disease. Saturation recovery methods have the potential to provide a more accurate measurement of T1 that is less sensitive to MT as well as other factors. Saturation recovery techniques are, however, noisier and somewhat more artifact prone and have not demonstrated the same level of reproducibility at this point in time.

This review article focuses on the technical aspects of key T1-mapping methods and imaging protocols and describes their limitations including the factors that influence their accuracy, precision, and reproducibility.

## Introduction

The longitudinal relaxation time constant (T1) of the myocardium is altered in various disease states due to increased water content or other changes to the local molecular environment. Changes in both native T1 and T1 following administration of gadolinium (Gd) based contrast agents are considered important biomarkers and multiple methods have been suggested for quantifying myocardial T1 in vivo [1]. Characterization of the native T1 of myocardial tissue may be used to detect and assess various cardiomyopathies while measurement of T1 with extracellular Gd based contrast agents provides additional information about the extracellular volume (ECV) fraction. The latter is particularly valuable for more diffuse diseases that are more challenging to detect using conventional late gadolinium enhancement (LGE). A number of recent papers have highlighted applications of T1-mapping in cardiovascular magnetic resonance (CMR) and their potential for detecting diffuse cardiomyopathies. Both T1 and ECV measures have been shown to have important prognostic significance [2,3].

Late gadolinium enhancement (LGE) is currently the primary tool for tissue characterization in CMR and provides excellent depiction of myocardial infarction (MI) and focal scar, and has become an accepted standard for assessing myocardial viability [4]. LGE is also useful for detecting and characterizing fibrosis that is “patchy” in appearance, e.g. as seen in non-ischemic cardiomyopathies such hypertrophic cardiomyopathy (HCM) [5]. Diffuse myocardial fibrosis is, however, more difficult to distinguish using LGE since the myocardial signal intensity may be nearly isointense and may be globally “nulled” thus appearing to be normal tissue [6]. Alternatively, quantitative measurement of myocardial T1 following administration of an extracellular Gadolinium-based contrast agent has been shown to be sensitive to increased extracellular volume associated with diffuse myocardial fibrosis. However, a single post-contrast T1 measurement has limitations due to a variety of confounding factors [7,8] such as gadolinium clearance rate, time of measurement, injected dose, body composition, and hematocrit. These factors cause a significant variation in post-contrast T1 making it difficult to distinguish diseased and normal tissue based on absolute T1 values alone. Pre-contrast T1 varies with water content and may be elevated in cases of diffuse myocardial fibrosis. Pre-contrast T1 also varies significantly with field strength [9]. Direct measurement of extracellular volume (ECV) was initially developed for quantifying the myocardial extracellular fractional distribution volume [10] and has been proposed as a means for detection and quantification of diffuse myocardial fibrosis [6,11-18]. This approach is based on the change in T1 following administration of an extracellular contrast agent and circumvents the limitation of a single post-contrast T1 measurement in detecting a global change in T1. Myocardial ECV is measured as the percent of tissue comprised of extracellular space, which is a physiologically intuitive unit of measurement and is independent of field strength. ECV has been shown to correlate with collagen volume fraction in some diseases [12,13].

Native T1-mapping as well as ECV mapping is currently being explored as a diagnostic tool for a wide range of cardiomyopathies. Native T1 changes are detectable in both acute and chronic MI [19,20], and may be used to characterize the edematous area at risk [21-23]. Elevated native T1 has also been reported in a number of diseases with cardiac involvement: myocarditis [24], amyloidosis [25], lupus [26], system capillary leakage syndrome [17], and decreases in native T1 have been associated with Anderson Fabry disease [27], and high iron content [28,29].

In general, methods for measuring myocardial T1 consists of three components: 1) a perturbation of the longitudinal magnetization (i.e. an inversion or saturation), 2) an experiment to sample the relaxation curve as the longitudinal magnetization returns to its original level, and 3) a model used to fit the sampled curve and extract the myocardial T1. This paper focuses on the technical aspects of key methods and imaging protocols and describes their limitations and the factors that influence their accuracy, precision, and reproducibility. The accuracy and precision of these measurements affect the detection and quantification of abnormal myocardial tissue.

The sensitivity for detecting abnormal elevation of T1 and ECV is fundamentally limited by the precision of T1 estimates, which is a function of the number and timing of measurements along the inversion- or saturation-recovery curve, the signal-to-noise ratio (SNR), the tissue T1, and the method of fitting. Other factors that may not be random may also introduce errors that further limit the reproducibility. To successfully optimize imaging protocols, it is beneficial to understand the factors that influence the measurement accuracy.

A consensus statement by the T1-mapping working group [1] provides a general framework of recommendations. Well-controlled and optimized protocols are key to reproducibility, which is particularly important in applications aiming at detection of subtle fibrosis and pre-clinical disease. It is important to understand artifact mechanisms in parametric mapping which may be less familiar than conventional CMR artifact mechanisms.

### Brief history of methods for T1-mapping in the heart

Methods for measuring myocardial T1 were initially based on region of interest (ROI) analysis rather than pixel-wise parametric maps. Inversion recovery images at different inversion times were acquired with multiple breath-holds [30] or inversion recovery cine protocols were used as a means of acquiring data in a single breath-hold [31]. These early methods were ROI based schemes and were not suitable for pixel-wise mapping. Pixel-wise T1-mapping first appeared on the scene with the introduction of the MOLLI imaging strategy [32], which propelled the use of T1-mapping in CMR and inspired many new methods. MOLLI is widely used today with some protocol optimization and other adaptations. A shortened breath-hold adaptation with conditional curve fitting (ShMOLLI) [33] was proposed as a means of mitigating heart rate dependence as well as shortening the breath-hold. Further protocol optimization has been aimed at shortening the breath-hold and optimizing precision [15,34]. Motion correction was developed to mitigate respiratory motion for subjects with poor breath-holding [35] and phase sensitive inversion recovery reconstruction with motion correction further improved image quality [36]. A number of publications analyzed the accuracy of T1 measurements [32,33,37-42] leading to a better understanding of the influence of various protocol parameters on T1-measurement errors. Saturation recovery methods that were developed initially for T1-measurements during first pass contrast enhanced perfusion (SAP-T1) [43] have been recently adapted for T1-mapping using SSFP readout (SASHA) [44] as a means of mitigating the T1-underestimation in MOLLI. Even more recently, hybrid schemes have been proposed that incorporate both inversion and saturation recovery methods (SAPPHIRE) [45]. ECV measurements were initially introduced using ROI based measurement [10-12] and pixel-wise ECV mapping was later introduced [16,34]. Improvements to T1- and ECV-mapping are continuously introduced, and this review provides a snapshot of the current state-of-the-art from our perspective.

This paper reviews the basic concepts behind the widely used MOLLI and SASHA acquisition strategies for T1-mapping, followed by a review of factors influencing accuracy and precision. Discussion includes a description of other limitations and a summary of pros and cons of various protocols.

## Methods

### Acquisition strategies and protocols

The currently used protocols for T1-mapping in the heart (Table 1) are based on inversion (IR) or saturation recovery (SR). Images are acquired at multiple time points on the recovery curve, and pixel-wise curve fitting is performed to estimate the relaxation time parameter to produce a pixel-map of T1. Images are generally acquired at the same cardiac phase and respiratory position to eliminate tissue motion. Although initial implementation involved multiple breath-holds, current methods generally use single breath-hold protocols with single shot 2D-imaging. To achieve higher spatial resolution and/or 3D imaging segmentation may be required.

**Table 1:** Widely used inversion and saturation recovery methods for T1-mapping in the heart

		
Inversion Recovery (IR)	Multiple breath-hold FLASH	[30]
	MOLLI	[32]
	ShMOLLI	[33]
	Modified MOLLI Protocols	[15,16,34,39]
Saturation Recovery (SR)	SAP-T1	[43]
	SASHA	[44]
Combined IR/SR	SAPPHIRE	[45]

**Figure 1:**
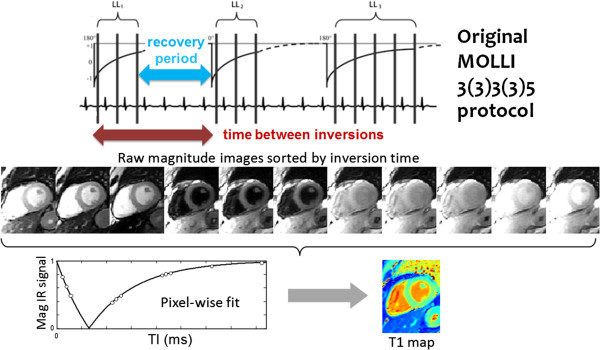
**MOdified Look-Locker Inversion Recovery (MOLLI) scheme for T1-mapping in the heart **[32]**.** The original protocol employed 3 inversions with 3, 3, and 5 images acquired in the beats following inversions, and 3 heart beat recovery periods between inversions, referred to here as 3(3)3(3)5. All images are acquired at the same delay from the R-wave trigger for mid-diastolic imaging. Curve fitting is performed on a pixel-wise basis using the actual measured inversion times.

The original scheme known as the MOdified Look-Locker Inversion Recovery (MOLLI) proposed by Messroghli, et al. [32] is illustrated in Figure 1. For each inversion, the MOLLI method samples the IR curve at multiple inversion times using single shot imaging spaced at heart beat intervals. Multiple inversions are used with different trigger delays in order to acquire measurements at different inversion times to sample the IR curve more evenly. Recovery periods are needed between the inversions to ensure that samples from the different inversions are from the same recovery curve, i.e., each inversion starts at the same initial magnetization. The T1-map precision is related to the number and position of samples along the IR curve, and accuracy of the signal model is also affected by the sampling strategy due to the influence of the readout on the apparent recovery.

**Figure 2:**
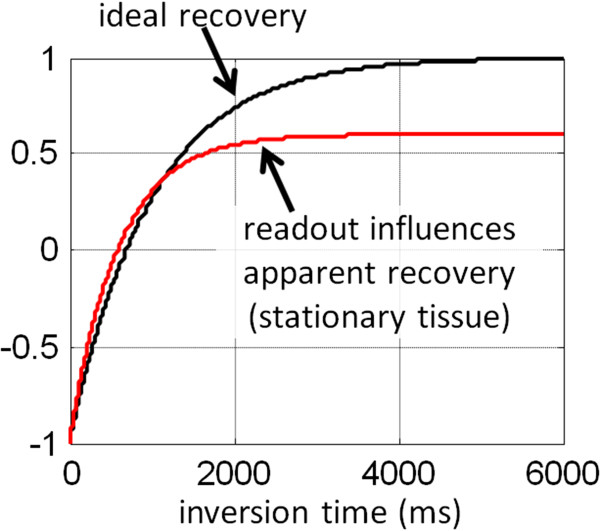
The apparent inversion recovery (T1*) is influenced by the SSFP readout. The effective inversion recovery is fit using a 3-parameter model, and the T1 is estimated using the so-called Look-Locker correction.

The MOLLI method uses a steady state free precession (SSFP) readout. The readout drives the IR to recover more quickly and reaches a steady state that is less than the equilibrium magnetization (M0). The effect of the readout (Figure 2) is an apparent recovery time referred to as T1* which is less than the actual longitudinal recovery time, T1, which is the desired tissue parameter. As a result of the influence of the readout, the inversion recovery curve follows a 3-parameter exponential signal model, S(t) = A – B exp(−t/T1*), where t represents the inversion time, and T1* is the apparent T1. The measured values may be fit to the 3-parameter model to estimate A, B, and T1* which may be used to approximate T1 ≈ T1* (B/A – 1). The derivation for the so-called “Look-Locker” correction factor (B/A – 1) is based on a continuous readout using Fast Low Angle SHot (FLASH) [46]. Despite the fact that the MOLLI uses a gated SSFP readout, the signal model behaves as a 3-parameter model where the Look-Locker correction is reasonably effective at low readout excitation flip angles.

The analytic relationship between T1* and T1 for SSFP has been derived for continuous SSFP under somewhat idealized conditions such as ideal slice profile [47]. Useful analytic derivations for gated SSFP with realistic slice profiles have not been developed due to complexity. Bloch simulations may be used to calculate the error inherent in this approximation [38] and to gain insight into the sensitivity of various protocol parameters and design variables. The influence of various parameters on accuracy is provided in following Sections.

A number of protocol modifications (Table 2) have been proposed to shorten the acquisition duration or to improve the accuracy or precision [15,33,39,41]. In this paper, a shorthand nomenclature is used to label these protocols. The notation captures how many inversions (or saturations) are included in the experiment, how many images are acquired after each inversion, and how long the waiting period is between inversions. For example, a 3(3)3(3)5 protocol would indicate that there are a total of 3 inversions; 3 images are acquired (over 3 RR intervals) after the first inversion, this is followed by a waiting period of 3 RR intervals, then 3 images are acquired followed by another 3 RR waiting period, finally a third inversion after which 5 images are acquired. In an extension of this nomenclature, an “s” can be added to the intervals to indicate that images are acquired for a certain number of seconds and the waiting period is in seconds, i.e., 5s(3s)3s would indicate 2 inversions with acquisition of images for at least 5 s, followed by a recovery of at least 3 s, and a second inversion with images acquired for at least 3 s. Since number of ECG triggered images must be a whole number, the acquisition and recovery periods are rounded to the nearest multiple of the RR-period to ensure an adequate duration. In order to avoid acquiring too few images for low heart rates (<60 bpm), the sequence never acquires fewer than the specified number of images, i.e., 5 + 3 = 8 in this example. The recovery period is never less than the specified number of seconds. Acquiring and recovering with fixed minimum time periods helps gain independence of heart rate.

**Table 2:** Reported schemes for MOLLI sampling

		
MOLLI	3(3)3(3)5	[32](original publication)
	3(3)5	[16,39]
	5(3)3	[34]
	4(1)3(1)2	[15]
	2(2)2(2)4	[39]
	5(3s)3	[41,42]
	4(1s)3(1s)2	[41]
	5s(3s)3s	
	4s(1s)3s(1s)2s	
ShMOLLI	5(1)1(1)1 (with conditional fitting)	[33]

**Figure 3:**
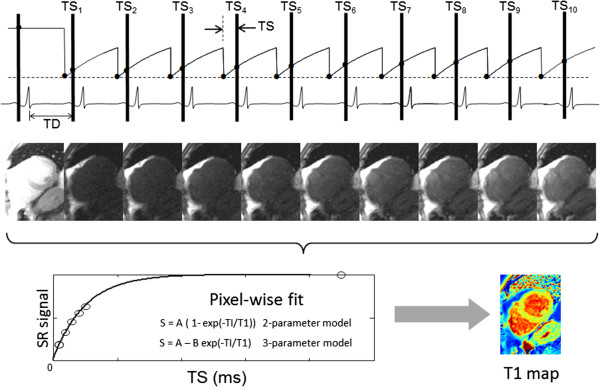
**SAturation recovery Single Shot Acquisition (SASHA) scheme for T1-mapping in the heart **[44]**.** A single image is acquired without saturation and used as the fully recovered measurement followed by a series of saturation recovery images at different saturation recovery times (TS_i_). All images are acquired at the same delay from the R-wave trigger for mid-diastolic imaging. Curve fitting is performed on a pixel-wise basis.

Saturation recovery (SR) is an alternative to inversion recovery that has gained renewed attention. Despite having a reduced dynamic range, saturation recovery has potential for improved accuracy. SR methods that use a saturation preparation for each measurement have the benefit that each measurement becomes independent of the others. By starting the recovery from a saturated state, the prior history is erased. Recovery periods between successive measurements are not required unless longer saturation recovery times are needed for fitting. The method known as SAturation recovery Single Shot Acquisition (SASHA) [44] is diagrammed in Figure 3. The SASHA method using SSFP readout is very similar to the earlier Short Acquisition Period - T1 (SAP-T1) method [43] which used a spoiled gradient recalled echo (GRE) readout.

The SASHA method acquires multiple time points on the SR curve and does a pixel-wise curve fit. In order to acquire a fully recovered image, an image is initially acquired prior to any saturation preparation, i.e., starting from the equilibrium magnetization. Images are acquired on successive heart beats using SR preparations with varying trigger delays. In the original proposed SASHA protocol there are 10 images acquired at saturation delays uniformly spaced over the RR-interval plus the initial fully recovered image which serves as an important anchor point for the curve fit. The order in which the various delays are acquired is not of significant importance for fitting assuming ideal saturation. Importantly, the SR curve recovers as T1 and is not influenced by the readout so that it is not shortened to an apparent T1* < T1 as in the case of MOLLI. Therefore, no correction is necessary, which eliminates the source of many inaccuracies of the IR based MOLLI scheme. Since the readout does not lead to an apparent T1*, a higher flip angle readout is possible which makes up for some of the lost dynamic range in using SR. The higher readout flip angle readout using SSFP with linear phase encode ordering does slightly alter the shape of the recovery curve causing an apparent bias, i.e., curve does not start at 0 for 0 delay. Thus, the otherwise 2-parameter signal model S(t) = A(1- exp(−t/T1)) for SR assuming ideal saturation, becomes a 3-parameter model S(t) = A – B exp(−t/T1). The 3-parameter model also absorbs any imperfection in the saturation efficiency due to the RF saturation pulse. However, the cost of estimating the additional parameter leads to a loss of precision. Therefore, as in all things, there is a trade-off between accuracy and precision in considering whether to use 2 or 3-parameter fitting, which is analyzed in more detail in the following. Although a center-out phase encode order in which the center of k-space is acquired first has the potential to completely remove the influence of the readout, the use of center out ordering with SSFP is problematic due to artifacts and the use of center-out FLASH is associated with a significant loss of SNR.

The SASHA sampling scheme may be altered to acquire longer saturation delay measurements by allowing 1 or more heart beat recovery periods between saturations. However, measurements cannot be made during the recovery periods without distorting the curve, thus additional measurements reduce the overall SNR efficiency somewhat. Schemes that simply use a MOLLI strategy replaced with SR [48] incur the problems of an apparent T1* without gaining the main benefits of SR. A combined IR/SR approach known as SAturation Pulse Prepared Heart rate independent Inversion-REcovery (SAPPHIRE) [45] gains many of the benefits of IR and SR but still retain some of the problems associated with IR. Each method has its strengths and weaknesses in terms of accuracy, precision, and overall reproducibility, which are examined in greater detail in the following.

## Accuracy & precision

**Figure 4:**
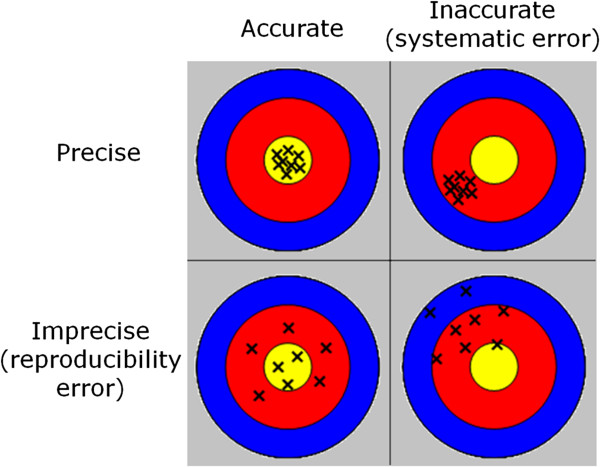
**Illustration of accuracy versus precision.** Accuracy refers to systematic errors, which create a bias, whereas, precision relates to the random component due to noise (http://www.jcmr-online.com/content/15/1/56/figure/F1).

The performance of quantitative methods may be assessed and compared in terms of accuracy and precision. Accuracy relates to systematic bias errors whereas precision relates to random errors due to noise (Figure 4). Other sources of variation that affect the reproducibility are “biases” that arise from a variety of influences that are not well controlled but are not random. These might include aspects such as dependencies on protocol parameters, artifacts, or effects such as partial volume.

### Accuracy: systematic errors and biases

Factors influencing the accuracy of T1-measurement using inversion and/or saturation recovery methods are listed in Table 3. These sources of error are divided into broad categories of: protocol parameters, sequence design, scanner adjustments, fit model, tissue characteristics, and patient related. The user can generally vary protocol parameters to some degree but must understand how any protocol changes might influence the T1-values. For instance, changing matrix size may influence the T1-estimate having an unintended consequence to reproducibility. The sequence design will generally influence the accuracy; the user must exercise caution when comparing data acquired between different versions of sequences or between different vendor platforms. Scanner adjustments of shim, center frequency, or transmit power level may have a strong influence on measurement accuracy unless the sequence is designed to be sufficiently robust to the expected variations. Curve fitting models and approaches as well as other image reconstruction steps may influence biases as well as precision. The tissue characteristics such as T2 or multiple compartments in exchange leading to magnetization transfer (MT) effects may strongly influence the measurement of T1 depending on the measurement technique. Patient related factors such as heart rate and respiratory motion may also affect the measurement. Sensitivity to some of these parameters is described in following subsections.

**Table 3:** Factors influencing the accuracy of T1-measurement using inversion and/or saturation recovery methods

		
**Protocol parameters**	Matrix size	• Underestimation in T1 depends on the protocol parameters
	Parallel imaging	
	Partial Fourier	• Precision depends on the sampling strategy
	Flip angle	
	Echo-spacing (BW & TR)	• Partial volume errors depend on the spatial resolution and slice thickness
	# images & acquisition strategy	
	Inversion times	
	Recovery times	
	Raw filter	
**Sequence design**	Slice profile	• T1 measurement accuracy is influenced by the sequence design
	Inversion pulse efficiency & BW	
	SSFP steady state run-up	
**Scanner adjustments**	Shim	• Off-resonance causes both regional and global underestimation of T1
	Center frequency adjustment	• Short z-FOV influences recovery time for inflowing blood
	B1 transmit ampl (flip angle)	• Scan to scan variation affects reproducibility
	z-FOV	
**Fit model**	2 vs 3 parameters	• Fitting additional parameters worsens precision
	Multi-fit MagIR vs PSIR	
**Tissue characteristics**	T2	• Tissue characteristics influence the apparent inversion recovery
	MT	
	Fatty infiltration	• Partial volume effects are an artifact and may contaminate measurements
	Flow	
**Patient**	Heart rate	• Loss of spatial resolution due to motion increases the partial volume problem
	Respiratory motion	

Many of the errors in the MOLLI scheme which uses inversion recovery with SSFP readout are a result of the approximation of the so called Look-Locker correction which attempts to correct for the fact that the apparent T1-recovery time is less than the true recovery time. The apparent T1* shortening is T2-dependent as a consequence of the SSFP behavior [33,37-39,44]. This error leads to a series of dependencies such as heart rate dependence and sensitivity to off-resonance, which will be described in the following paragraphs. Interdependence of parameters makes it difficult to neatly describe the performance. In this discussion, we begin with a set of nominal parameters and examine deviations of a single parameter such as heart rate, off-resonance, or flip angle to gain insight into the sensitivity of that specific parameter.

The calculation of T1-errors in this article is based on waveform level Bloch-simulations and curve fitting using the following MOLLI and SASHA protocols. Existing studies in the literature rely heavily on simulations but are difficult to compare directly as a result of different assumed protocol parameters (e.g., slice profile) or methodology of simulation. To simplify comparisons, all analysis presented here use common methods and assumptions. The SSFP readout used a 480 μs low time-bandwidth product Hamming weighted sinc pulse with ≈ 8 mm slice thickness, and TR = 2.8 ms (bandwidth 1085 Hz/pixel), and 5 pulses with linear ramp flip angle to catalyze toward steady state. The matrix (256×144) assumed parallel imaging with factor 2 acceleration, separate reference lines, and partial Fourier factor of 7/8 in the phase encoding direction. The actual number of phase encodes was 63 with center at line 27. MOLLI used a tan/tanh adiabatic inversion [40] with 2.56 ms duration, and SASHA used an adiabatic BIR4-90 with 5.12 ms duration. Excitation flip angles were 35° and 70° for MOLLI and SASHA, respectively, unless otherwise noted. MOLLI used a minimum TI of 100 ms, and TI increment of 80 ms. SASHA acquired a fully recovered image plus 10 additional images acquired with saturation times spaced uniformly over the RR interval with minimum “inversion” time of 100 ms. MOLLI used PSIR 3-parameter curve fitting, and SASHA used both a 3-parameter fitting as originally proposed [44] as well as 2-parameter fitting as introduced here. Other parameters such as MOLLI sampling scheme (e.g. 5(3s)3), tissue, off-resonance, and heart rate were variable as indicated. In-vivo measurements were acquired on both 1.5 T (Siemens Magnetom Aera) and 3 T (Siemens Magnetom Skyra) MRI clinical scanners. In-vivo data presented here was acquired under a study protocol approved by the local Institutional Review Board and all subjects gave written informed consent.

### Sensitivity to T2

**Figure 5:**
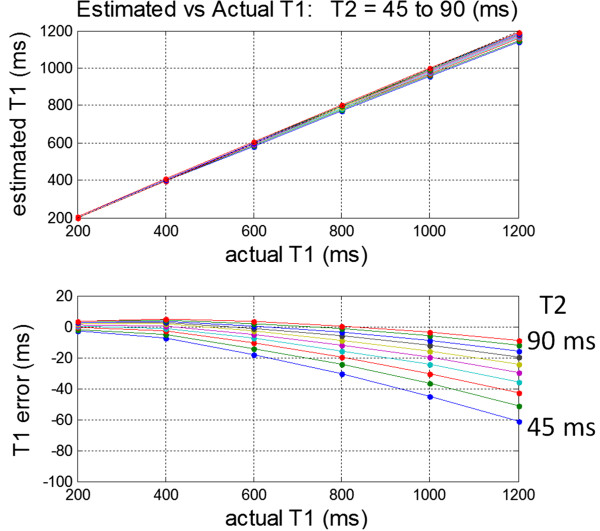
**The estimate of T1 using SSFP based MOLLI 5s(3s)3s is sensitive to T2 with increased underestimation error at lower values of T2 which results in a T1 map which has a small degree of T2 weighting.** For native myocardium, an increase of 100% in T2 from 45 to 90 ms results in an increase in the apparent T1 of approximately 4%.

The apparent inversion recovery is influenced by T2 using an SSFP readout leading to a T2 dependent error in the estimate of T1 (Figure 5) [38]. The resultant T1-map will have a slight T2 weighting. The imperfect inversion efficiency of the adiabatic RF preparation also contributes to a T2 dependent error that is minimized by using a short duration inversion pulse [40]. In the case of edematous tissue with elevated T1 and T2, the apparent T1 elevation will be increased by a slight amount thereby improving the detectability. Saturation recovery methods such as SASHA using 3-parameter fitting do not experience influence due to the SSFP readout and unlike MOLLI are therefore not sensitive to T2 [44]. SASHA using 2-parameter fitting has a slight T2 sensitivity.

### Influence of off-resonance

Off-resonance is well known to cause banding artifacts using SSFP readout. It is not well appreciated, however, that a significant error in T1 may result at relatively small off-resonance frequencies that are well within the region without banding artifacts [42]. Regional variations due to the inability to completely shim the B0-field variation around the heart may appear as regional variation in T1 that is artifactual. Although shimming problems are more of an issue at higher field strengths, the variation at 1.5 T is significant enough to be of concern particularly if it is not recognized. In a small study, the off-resonance frequency (after localized shimming) in the LV was measured across n = 18 subjects to have a mean value of 20.3 ± 13.0Hz at 1.5 and 15.4 ± 29.3Hz at 3 T, and to have maximum off-resonance of 61.8 ± 15.5 Hz at 1.5 T and 125.0 ± 40.6 Hz at 3 T [42]. At 1.5 T, off-resonance greater than 80 Hz was observed in 4 of 18 subjects, which resulted in more significant T1 errors (> 3%) that could be erroneously interpreted as subtle regional variation of apparent T1 [42]. Previous analysis of off-resonance related T1 errors in MOLLI [38] considered a smaller off-resonance (<50 Hz) frequencies which lead to minor errors. Previous analysis of off-resonance in SASHA only considered 3-parameter fitting [44]; here the simulations are expanded to include 2-parameter fitting for SASHA.

**Figure 6:**
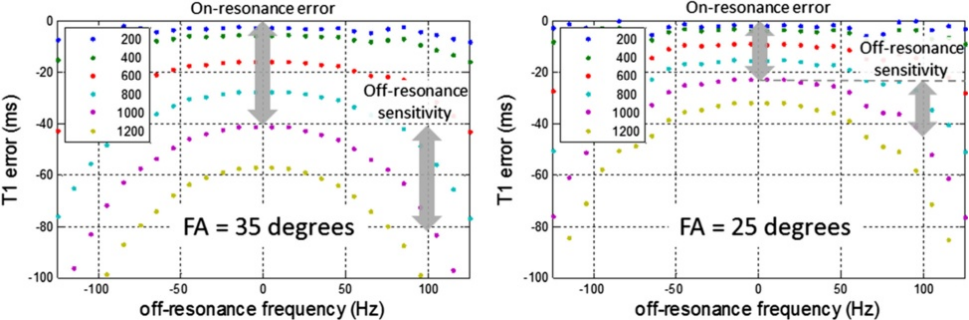
**Simulated off-resonance response of MOLLI for 5s(3s)3s protocol with TR = 2.8 ms using FA = 35 (left) and 25 (right) at various T1’s for myocardial T2 = 45 ms.** Using a lower flip angle (FA) trades SNR (precision) for improved accuracy and reduced off-resonance sensitivity. For T1 = 1000 ms, sensitivity to off-resonance over ±100 Hz is 40 ms and 25 ms for FA = 35° and 20°, respectively.

**Figure 7:**
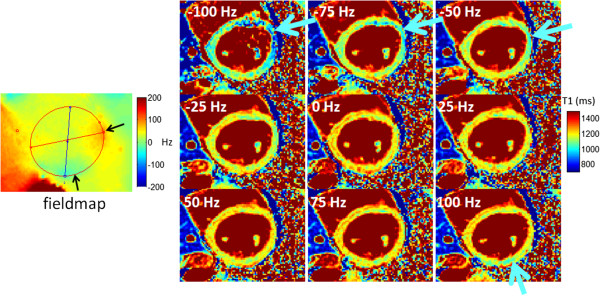
**T1-maps acquired at different center frequencies using MOLLI 5s(3s)3s at 3 T.** Despite the use of a 2nd order shim in a local volume around the heart, off-resonance variation across the heart, as seen in the field map (left), leads to an artifactual local variation in the apparent T1 as indicated by arrows [42]. (adapted from http://www.jcmr-online.com/content/15/1/63/figure/F5 and http://www.jcmr-online.com/content/15/1/63/figure/F6).

**Figure 8:**
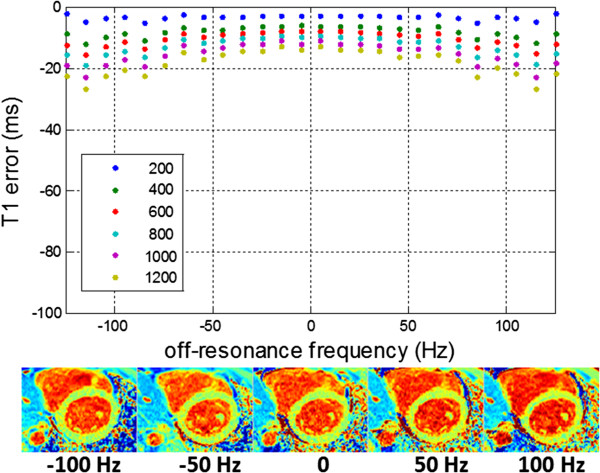
**Simulated off-resonance response of SASHA using a 2-parameter fit, BIR4-90 saturation pulse, TR = 2.8 ms, FA = 70° at various T1’s for myocardial T2 = 45 ms.** Using a 3-parameter fit has virtually no off-resonance sensitivity (< 10 ms error across ±100 Hz).

The sensitivity of a typical MOLLI protocol is shown in Figure 6 for a range of T1 values and 2 readout flip angles. Reducing the flip angle will reduce the off-resonance related error at the expense of a reduction of SNR causing a loss of precision, i.e. noisier maps. An in-vivo example at 3 T (Figure 7) illustrates how a variation in shim appears as a variation in apparent T1 when there is center frequency adjustment error. The SASHA method is less sensitive to off-resonance. The 3-parameter fit is highly insensitive to off-resonance [44]. The 2-parameter fit is sensitive to saturation efficiency thus it is important to use a saturation pulse that achieves a high degree of saturation over the expected bandwidth. The off-resonance sensitivity of SASHA with a 2-parameter fit using an optimized BIR4-90 design achieves a variation of < 10 ms over ±100 Hz (Figure 8).

### Influence of heart rate

**Figure 9:**
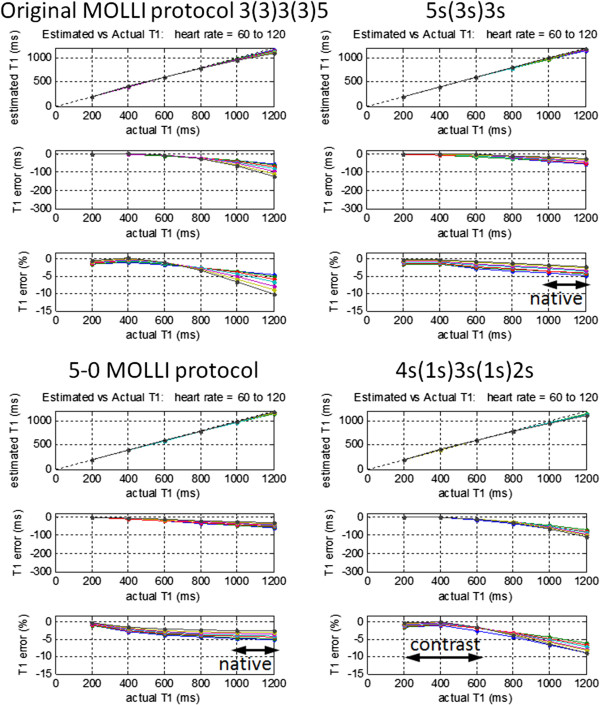
**Influence of heart rate on estimate of myocardial T1 for various MOLLI protocols with T2 = 45 ms, and flip angle = 35°.** The original MOLLI protocol (top left) had a significant sensitivity to heart rate which may be reduced by increasing the time between inversions as in 5s(3s)3s protocol (top right), or by discarding samples for longer T1 as done in a ShMOLLI conditional reconstruction, approximated by 5(0) sampling for longer T1-values (bottom left). For lower values of T1 associated with Gd contrast, it is possible to improve accuracy using a 4s(1s)3s(1s)2s sampling scheme without incurring significant heart rate dependence (bottom right).

The influence of heart rate on the accuracy of T1 was recognized in the original MOLLI publications [32,37] and was a subject of considerable interest at that time since the original MOLLI protocol exhibited a large sensitivity to heart rate for long T1 values. The heart rate sensitivity of MOLLI has been significantly reduced by modification of protocols (Figure 9) to the point where it is of much less concern. There are 2 primary factors that affect the MOLLI heart rate sensitivity, a) the time between inversions, and b) the influence of the SSFP readout during each inversion recovery. The largest contributing factor to the original MOLLI heart rate sensitivity was the time between inversions. This factor can be mitigated by using a single inversion, or by increasing the time between inversions.

The time between inversions may be increased by simply changing the order of inversions used in the sampling strategy. The original sampling strategy 3(3)3(3)5 acquired 11 images in 17 heart beats with 3 inversions. The spacing between inversions was 6 heart beats which meant that at higher heart rates the magnetization was not fully recovered for subsequent inversions. A 5(3)3 strategy which acquires 8 images in 11 heartbeats using 2 inversions has significantly improved heart rate sensitivity by increasing the spacing between inversions from 6 to 8 beats [34]. This protocol evolved further to modify the recovery to be determined in seconds [41], 5(3s)3, and subsequently both acquisition and recovery to be determined in seconds (introduced here), 5s(3s)3s to ensure more complete recovery at high heart rates (> 60 bpm).

An alternative strategy to mitigate the heart rate sensitivity known as ShMOLLI [33] acquires using a 5(1)1(1)1 sampling scheme and performs conditional processing to discard the latter measurements for long T1 at high heart rates. In this scheme, 7 images are acquired in 9 heart beats with 3 inversions. For pixels with long T1 measured with short RR interval the data is re-fit using only the first 5 measurement of the 1^st^ inversion. This mitigates a large source of heart rate sensitivity, although there is a significant loss of precision associated with discarding data.

By eliminating the problem with multiple inversions at high heart rate, there still remains a few percent heart rate sensitivity due to the influence of the SSFP readout. This may be further reduced by decreasing the SSFP readout excitation flip angle at the expense of SNR. Note that it is possible to improve the accuracy for the lower range of T1s associated with use of Gd contrast agents (200–600 ms) by selecting a protocol with better sampling strategy such as 4s(1s)3s(1s)2s. This will also improve the measurement precision for short T1 as discussed later.

It is worth remarking that a number of early studies did simulations and phantom measurement over a range of T1 values > 2000 ms to account for blood as well as myocardium. While this is relevant for measurement in stationary phantoms, it turns out that the blood flow eliminates the beat-to-beat influence of the readout and therefore completely alters the error mechanism in T1 inversion recovery measurements. For this reason we believe that the estimation of T1 in flowing blood should be treated separately from the myocardium and long T1 values associated with blood are not relevant in the discussion of heart rate sensitivity.

### Influence of flip angle

**Figure 10:**
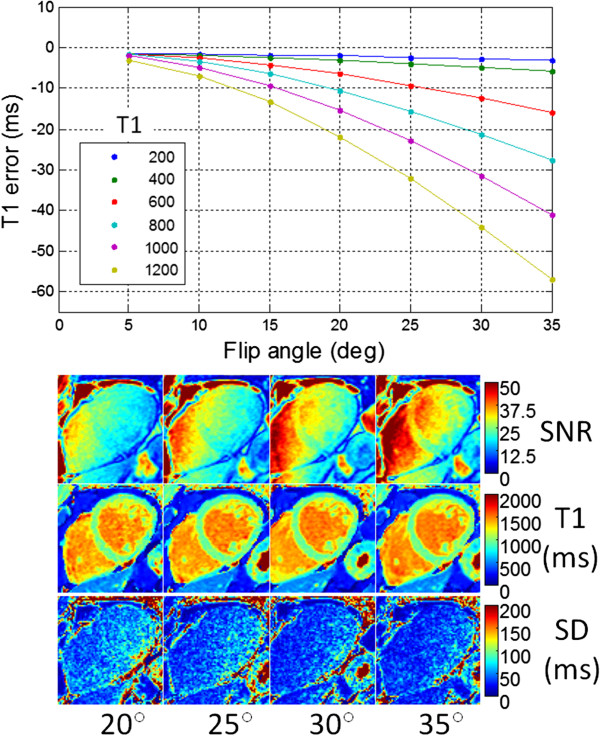
Sensitivity of myocardial T1 estimate using MOLLI 5s(3s)3s to excitation flip angle for various T1 values which has increasing T1 underestimation for increasing flip angle (top graph) and in-vivo examples for native myocardium at 1.5 T showing SNR maps, T1-maps, and standard deviation (SD) maps for flip angles of 20°-35° showing trade-off of SNR and precision.

The transmit flip angle will affect both the T1-measurement accuracy of MOLLI on-resonance as well as the off-resonance behavior and heart rate sensitivity as described. Flip angle also affects the SNR. There is increasing T1 underestimation of the myocardial T1 estimate for increasing flip angle using MOLLI shown in Figure 10 for various T1 values. In-vivo examples of T1-maps and corresponding maps of SNR and T1-measurement precision (standard deviation) illustrate this trade-off (Figure 10).

The transmit flip angle will vary due to the accuracy of transmitter calibration and will vary spatially due to inhomogeneity of the B1+ field. Variation in transmit flip angle across the heart is estimated to be 25% at 1.5 T, so this issue is not unique to higher field strengths. The variation in flip angle affects both the SSFP readout and the IR/SR preparation. The influence of the flip angle error due to SSFP readout is approximately a couple of percent. For instance at a T1 = 1200 ms, if there is a variation in flip angle from 28 to 35 degrees, the error will range from −40 to −60 ms, or an apparent variation in T1 of 20 ms (1.7%).

The SNR is related to the steady state magnetization, which varies with flip angle. The transverse magnetization in the native myocardium for the fully recovered image is almost double for SASHA using a 70° SSFP readout compared with MOLLI with 35° SSFP readout which helps to compensate to some extent for the loss inherent in SR compared with IR.

The performance of the IR and SR preparation pulses must be robust to the expected variation of B1+ field or the error may be potentially quite large. Adiabatic inversion and saturation pulses may be designed for this purpose to ensure that the sensitivity to flip angle is minimized [40] as discussed next.

### Influence of non-ideal inversion efficiency or saturation

**Figure 11:**
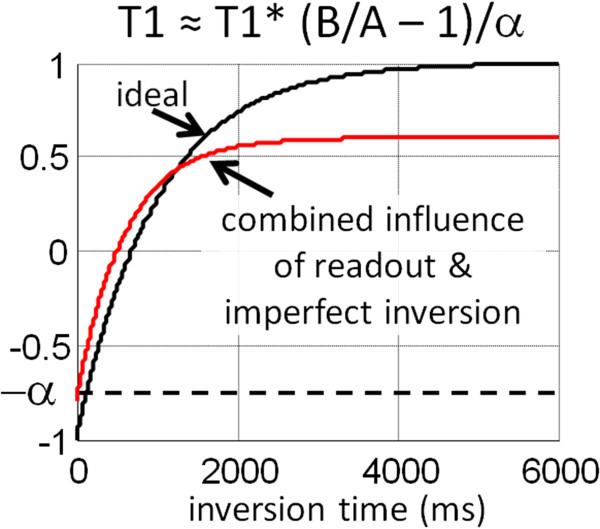
Imperfect inversion combined with the influence of SSFP readout alters the apparent T1* of the myocardium.

Adiabatic inversion pulses used to mitigate inhomogeneity of transmit B1 field strength do not achieve perfect inversion as a result of transverse relaxation (T2) during the pulse [40]. Imperfect adiabatic inversion leads to an error in estimating T1 (Figure 11) since the Look-Locker correction (B/A-1) of the apparent T1* assumes ideal inversion. Furthermore, the inversion efficiency may lead to a T2-dependent error in the T1 estimate. An optimized pulse design [40] with improved inversion efficiency can reduce this error as well as achieve a reduced T2-dependence. Empirical correction (T1 ≈ T1*(B/A-1)/α) for imperfect adiabatic inversion (α) may be used to further improve T1-measurement accuracy.

Saturation recovery methods rely on a high degree of saturation to achieve accurate estimates of T1 [44]. Methods such as SASHA are less sensitive to the degree of saturation if they use a 3-parameter signal model, which can absorb the non-ideal saturation to some extent along with the effect of the readout [44]. However, the 2-parameter model is highly sensitive to imperfect saturation. SR preparations that use a pulse sequel with crushers [44,49] are not adequate for use with 2-parameter fits. Using a BIR4-90 adiabatic pulse design optimized for saturation of the myocardium it is possible to achieve < 0.5% residual longitudinal magnetization over a 25% amplitude uncertainty and ±100 Hz uncertainty due to B0 variation.

### Fitting model and number of parameters

**Figure 12:**
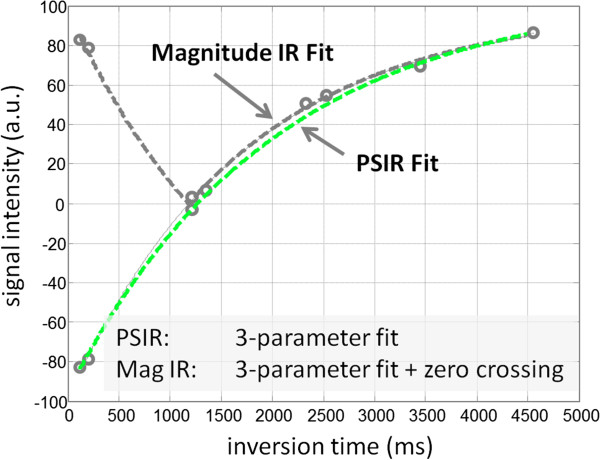
**Phase sensitive inversion recovery (PSIR) fitting uses a 3-parameter model, whereas magnitude IR fitting using a multi-fitting approach estimates 3-parameters plus the zero-crossing.** The multi-fitting magnitude IR fitting approach is prone to errors in estimating the zero-crossing in situations where the zero-crossing is close to the measured inversion times leading to a significant loss of precision for specific values of T1 and RR for a given protocol.

The signal model for inversion recovery based MOLLI with SSFP readout is a 3-parameter model. When magnitude detection is used in the image reconstruction the signal model becomes S_MAG_(t) = abs(A – B exp(−t/T1*)) and when phase sensitive detection (PSIR) is used becomes S_PSIR_(t) = A – B exp(−t/T1*). In the original proposed MOLLI scheme a multi-fit magnitude fitting approach was used in which the zero-crossing was determined by a procedure that performed a PSIR fit to the measured data with assumed zero-crossing time and appropriate signs, and then finding the value for zero-crossing that minimized the power in the residual fit errors. This approach is less sensitive to initial conditions than a direct magnitude fit. However, estimating 3-parameters plus the zero-crossing is a 4-parameter fit nonetheless and is therefore more prone to errors than PSIR fitting (Figure 12) [36]. The increased random error for multi-fit magnitude IR can be up to 30% for current protocols depending on the HR and T1. Furthermore, PSIR fitting maybe used directly after contrast administration when the blood pool Gd concentration is high, which can cause problems for multi-fit magnitude fitting methods at low T1.

Saturation recovery with ideal saturation is still based on a 3-parameter model (original proposed SASHA method) due to the influence of the readout [44]. Here, we also consider using a 2-parameter fit with SASHA. As shown in the simulations, this can greatly reduce the random error but introduces susceptibility to biases caused by imperfect saturation and due to influences of the SSFP readout. While the 3-parameter model is most accurate, the 2-parameter model underestimates the T1 by approximately 3-4% even with ideal saturation.

### Blood flow

**Figure 13:**
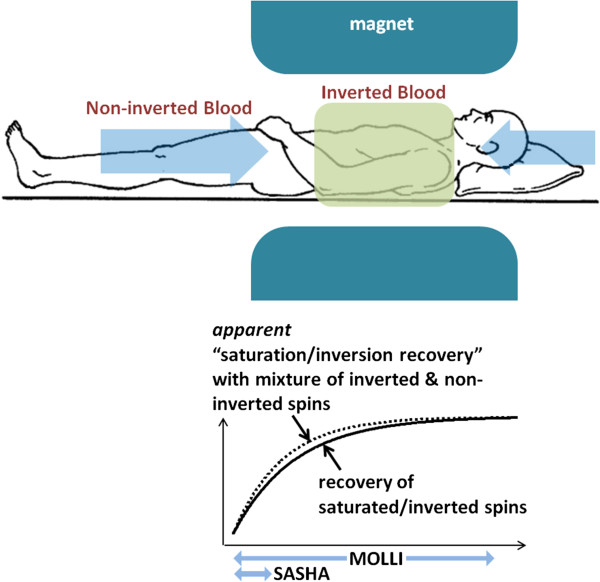
Mixing of non-inverted blood with inverted blood may alter the apparent inversion or saturation recovery.

**Figure 14:**
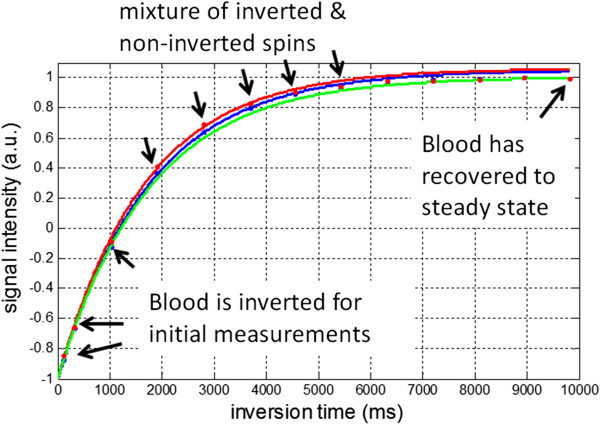
**In-vivo inversion recovery in blood illustrating in-flow of non-inverted blood.** Fit to RV (red) and LV (blue) blood pool measurements for inversion times up to 5 seconds, and fit to LV blood (green) for measurements up to 1 second and last measurement at 10 seconds. Fits for measurements up to 5 sec (blue and red) underestimate the blood T1 due to the mixture of inverted and non-inverted spins, which flow in from outside the inversion volume.

There are a number of key differences between the blood and myocardium. The blood T2 is 250 ms, whereas the myocardial T2 is approximately 45 ms. The longer T2 results in a more ideal inversion efficiency [40], as well as reduced influence due to the SSFP readout. The MT effect in blood is considerably lower than in the myocardium. Finally, the blood is flowing. There has been considerably less reported on the accuracy of T1 measurement in the blood pool in the presence of flow. Here, we share our perspective and initial experience on this subject. The flow of blood has 2 effects. Firstly, from beat to beat the blood is moving and mixing such that the slice selective SSFP readout from a given beat does not influence the next. As a result the apparent inversion recovery in the blood is simply T1 rather than T1* and does not require a Look-Locker correction. Although the measured correction factor (B/A-1) in the blood is close to 1 in this case, it may deviate due to imperfection in the inversion and due to MT. Secondly, the flow of non-inverted blood from the head and legs outside the magnet (or z-FOV) will mix with the inverted flow and cause an apparent shorter T1 (Figure 13). The MOLLI method is more sensitive to inflowing blood since it samples the recovery curve for several beats following the inversion, whereas the SASHA method samples the recovery in the 1^st^ RR following saturation before the non-saturated blood has flowed in. The initial sample following the non-selective inversion is not influenced by the in-flow of blood from outside the inversion volume as are samples at long inversion time that have reached steady. Samples that follow the initial heartbeat after inversion and prior to steady state may be contaminated, particularly in the RV (Figure 14) that experiences in-flow sooner. As a result, fitting the first few samples can lead to an artifactual difference in T1 observed in the LV vs RV. By acquiring for a longer period a more accurate estimate is possible.

The value of blood T1 is used mainly for calibrating the extra-cellular volume (ECV) fraction [10-12,15,16,34]. Fortunately, the in-flow effect mainly affects the longer native T1 and is much less important for the measurement of T1 with contrast. The error in ΔR1 used for ECV calculation is less affected by errors in the pre-contrast T1. The acquisition of MOLLI images for a fixed time interval specified in seconds rather than a fixed number of beats ensures that the inversion recovery curve is sampled adequately (i.e., full recovery) even at high heart rates.

Due to the fact that the blood has different characteristics and is influenced differently than the myocardium, it is advantageous to apply different fitting procedures for myocardium and blood. Values for T1 of the blood are used primarily in the calculation of ECV. Although a single map is produced from a single experiment, blood T1 values used in ECV calculation may be estimated more precisely by fitting to measurements averaged over a ROI. A 3-parameter fit without correction maybe used since there is no significant beat-to-beat influence.

### Spatial resolution and partial volume

Spatial resolution is particularly important in T1-mapping. The T1-mapping methods assume that the voxel is comprised of a single tissue species, e.g., myocardium or blood, and not a mixture. It is not generally practical to fit for multiple species. Therefore, it is imperative to have adequate resolution to avoid partial volume effects. The boundary between myocardium and blood cavity may be significantly blurred due to through-plane effects of a relatively thick slice (≈8 mm), and will also be blurred in-plane due to the distortion of the imaging point spread function. Additional loss of resolution may occur due to cardiac motion during the imaging period particularly at high heart rates or for subjects with RR variability, or due to residual uncorrected respiratory motion. The net result is that it may not be possible to make accurate measurements if the myocardial wall is thin. Caution must be exercised to avoid partial volume problems, and to recognize their potential to bias studies of subjects with thin walls or higher heart rates. It has been proposed to measure T1-values in the mid-wall region [3] or to define the myocardial border after eroding the contour between blood and myocardium [50]. Both of these recommendations are sound but still may result in bias due to contamination of the myocardial signal by blood.

**Figure 15:**
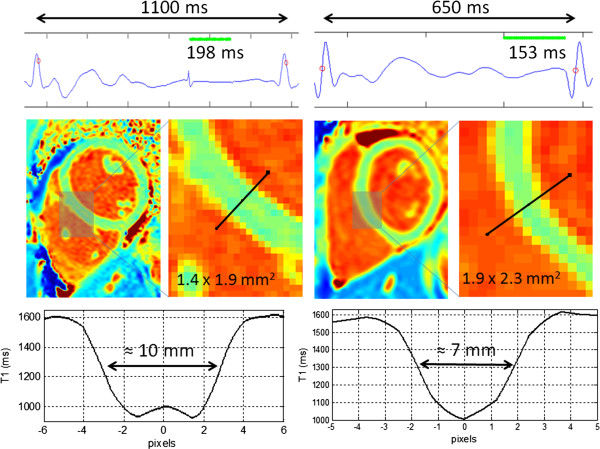
**Example of T1-maps in 2 subjects (a) (left) subject with heart rate of 58 bpm acquired using a MOLLI protocol with 256x144 matrix and (b) (right) subject with heart rate of approx.** 90 bpm using a 192 × 120 matrix. Although the interpolated maps are of good quality, the subject with higher heart rate and thinner wall has only about 3.5 pixels across the septum leading to a degree of partial volume error in ROI measurements.

Current protocols in use at our institution use a matrix size of 256x144 with 75% phase FOV for subjects with heart rate up to 90 bpm, and use a matrix of 192x120 for subjects with heart rates greater than 90 bpm in order to mitigate cardiac motion blur. Higher spatial resolution may be achieved using more aggressive parallel imaging. Example in-vivo maps (Figure 15) illustrate the in-plane resolution issue for thin walls at higher heart rates, which may lead to partial volume errors in quantitative measurements. These examples do not represent the best or worst case but are meant to illustrate a significant issue, which is often not appreciated when analyzing T1-maps. Thin wall atrial structures or RV wall present an even greater challenge. The Gibb’s ringing artifact in the image on the left of Figure 15 may be mitigated by use of raw filtering in the image reconstruction albeit at the expense of a slight loss of spatial resolution.

### Magnetization transfer

**Figure 16:**
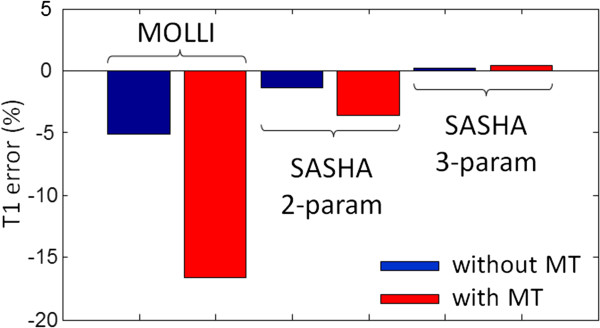
**Magnetization transfer (MT) significantly affects inversion recovery leading to an underestimation of native myocardial T1 using the MOLLI method.** Saturation recovery using higher SSFP readout flip angle causes an underestimation of SASHA using a 2-parameter fit. The 3-parameter fit SASHA is not influenced significantly by MT.

**Figure 17:**
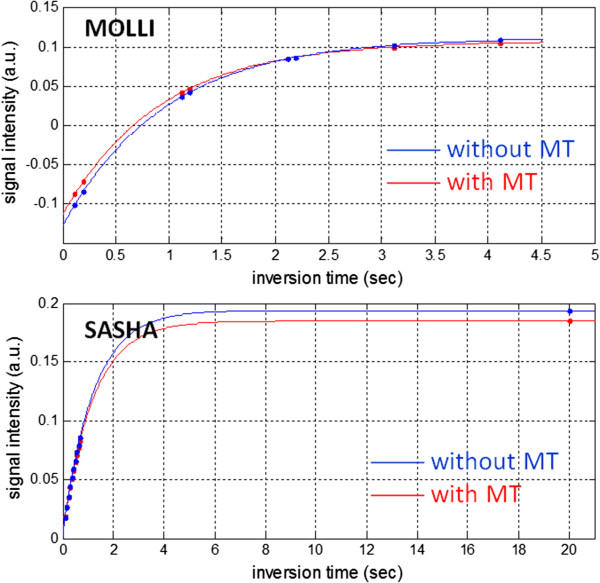
**The effect of magnetization transfer (MT) on the inversion recovery for native myocardial tissue using MOLLI (top) and on saturation recovery using SASHA (bottom).** MT changes the shape of the inversion recovery causing a shorter apparent T1*. MT has insignificant effect on the saturation recovery using SASHA with a 3-parameter fit.

Magnetization transfer (MT) has a significant effect on inversion recovery leading to apparent T1 estimates which are approximately 15% less than saturation recovery estimates in native myocardium [51] (Figure 16). Following the methodology used by Robson et al. [51], we have simulated the effect of MT to provide insight into the mechanism that alters the apparent inversion or saturation recovery. The primary reason for the shorter apparent inversion recovery appears to be that the so-called “bound” pool, which is in rapid exchange with the “free” pool, is not being inverted by the RF inversion pulse. This causes a rapid initial recovery that alters the shape of the inversion recovery curve (Figure 17). The Look-Locker correction does not correct for this effect. The SSFP readout (FA = 35°) using MOLLI also reduces the steady state value of the fully recovered image, which further contributes to the error. Saturation recovery methods such as SASHA are affected in a different manner and to a lesser extent. It is possible to saturate the bound pool so that the saturation recovery is less affected by MT. However, the influence of MT due to the SSFP readout using FA = 70° appears to be significant. Using a 3-parameter fit, this influence does not affect the T1-fit as shown by Robson, et al. [51] but comes at cost of significant precision loss. The MT of the SSFP readout does appear to affect the accuracy of SASHA using a 2-parameter fit (introduced here) leading to a shorter apparent T1, with underestimates of several percent.

Since the MT effect in inversion recovery is dominated by the MT tissue parameters more than protocol and scanner adjustments, it does not, in general, lead to reproducibility problems. As a result, the apparent T1 measured using MOLLI is highly reproducible despite significantly underestimating the T1 of the free pool [34,50]. The MT effect in the blood is greatly reduced since the bound pool fraction is much smaller [52-54]. The effect of MT with contrast enhanced myocardium is not well studied. Many myocardial T1-mapping methods are validated using phantoms in comparison with standards such as spin echo with a long repetition time, however, the MT effects for phantoms are generally negligible for low concentration gels typically used.

## Precision

The influence of random noise on the precision of various methods may be compared using Monte-Carlo methods. Other influences such as center frequency, which may in fact vary from study to study, will affect reproducibility but are not considered in the analysis of precision due to random noise. Precision depends on the SNR of the raw images and the number and location of samples along the recovery curve. Although equations for parameter error may provide insight into how the individual parameters affect precision [41], Monte-Carlo simulations provide a more straightforward means of comparing sampling strategies and protocols.

**Figure 18:**
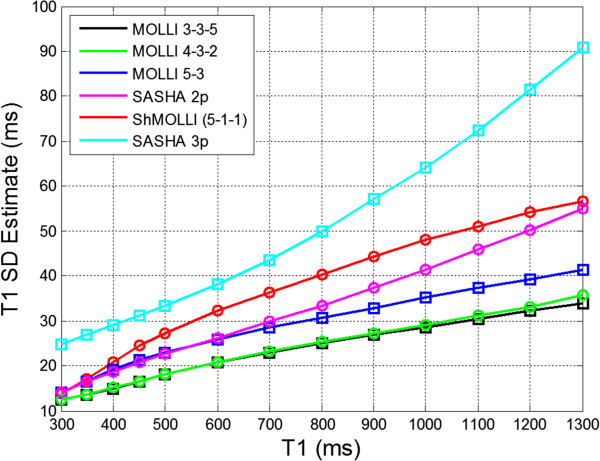
**Comparison of precision of various reported T1-mapping protocols using Monte-Carlo estimate of SD (n = 65536).** The heart rate was 60 bpm, and the SNR for MOLLI methods was 25, and for SASHA was 43 to account for the increased flip angle using the saturation recovery protocol.

The standard deviation (SD) of the T1 estimate increases with T1 for a given sampling scheme (Figure 18). The original MOLLI 3(3)3(3)5 protocol using 11 images has excellent precision albeit the accuracy degrades for long T1 values, particularly at higher heart rates. The MOLLI 4(1s)3(1s)2 with 9 images in a reduced breath-hold achieves similar precision and is used for shorter T1 values where the accuracy is not HR dependent. The MOLLI 5(3s)3 scheme with 8 images has excellent precision and may be used for native T1s without HR related bias but is not as optimal for shorter T1 values associated with contrast. The ShMOLLI scheme of conditional fitting [33] with 7 images sacrifices approximately 30% in precision due to the discarding of data without any improvement in accuracy. SASHA using 11 images acquired in the same breath-hold period as the MOLLI 5(3s)3 will have degraded precision but will have improved accuracy. Protocols that acquire images for a fixed time period such as MOLLI 5s(3s)3s have essentially the same precision as the 5(3s)3 at the 60 bpm where the RR = 1 s, but have improved precision at higher heart rates as there are more images acquired than 8. The precision loss for SASHA is approximately 35% using 2-parameter fitting and 125% using 3-parameter fitting compared to a MOLLI 5(3s)3 scheme with 3 parameter fitting. These calculations assumed an SNR of 25 for MOLLI inversion recovery schemes using a FA = 35°, and an SNR of 43 for SASHA due to the increased FA = 70° as measured for protocols with the same imaging parameters. The calculations for the MOLLI based schemes are based on phase sensitive inversion recovery (PSIR) reconstruction, which improves the precision by approximately 30% compared to multi-fit magnitude IR fitting for regions of T1, which have null times in the vicinity of the measured inversion times [36].

**Figure 19:**
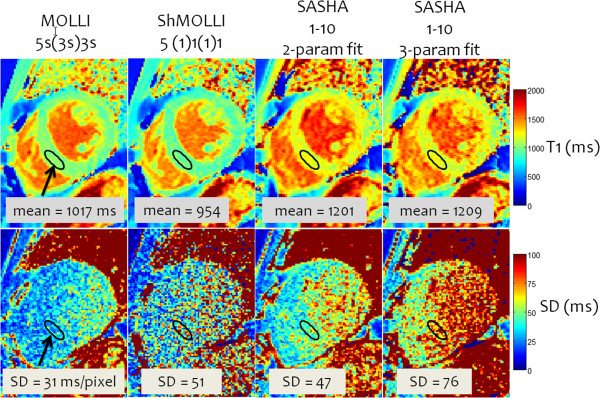
**Example In-vivo T1-maps and corresponding pixel-wise SD maps acquired using MOLLI 5s(3s)3s, ShMOLLI, and SASHA protocols using 2- and 3-parameter fitting.** Variation in SD across the heart is apparent due to variation in SNR from surface coil sensitivity roll-off. MOLLI has the best precision but underestimates T1 due to the approximate nature of the Look-Locker correction and due to magnetization transfer (MT). Note that SASHA with 2-parameter fitting has a small T1-underestimation; 3-parameter fitting is more accurate but has significant loss of precision.

In-vivo examples for native T1-maps (Figure 19) illustrate that the SD varies across the heart due to SNR variation resulting from surface coil sensitivity roll-off [41]. Myocardial SNR with the MOLLI protocol was found to be 43 ± 11 (m ± SD) in the septum and 22.8 ± 4.3 in the lateral wall measured in 20 subjects at 1.5 T using a voxel size of 1.4×1.9×6 mm^3^[41].

**Figure 20:**
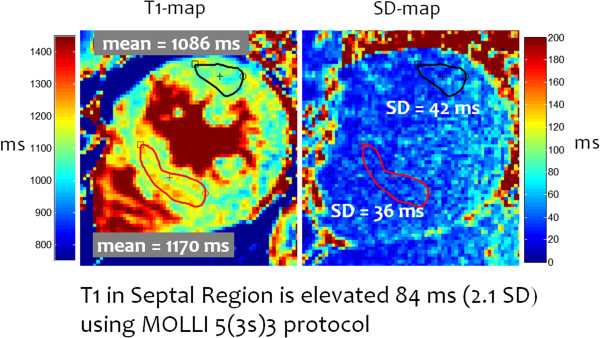
**Example native T1 and SD maps using MOLLI 5(3s)3 for a subject with HCM exhibiting focal native T1 abnormalities in the septal region corresponding to T1 elevation of 84 ms relative to the lateral wall representing an elevation of 2.3 SD on a pixel-wise basis (septal SD = 36 ms).** (adapted from http://www.jcmr-online.com/content/15/1/56/figure/F9).

Values for precision are presented as the SD per pixel, which is an important performance metric for pixel-wise mapping. Note however that the T1-precision will improve due to averaging when measuring T1 in a ROI. The SD will improve as sqrt(N_indep_) where N_indep_ is the number of independent pixels in the ROI, typically only about 50% of the pixels in the ROI are actually statistically independent due to factors such as interpolation, raw filtering, or partial Fourier acquisition. Example native T1 and SD maps (Figure 20) for a subject with HCM exhibiting focal native T1 abnormalities in the septal region corresponding to a T1 elevation of 84 ms relative to the lateral wall representing an elevation of 2.3 SD on a pixel-wise basis (septal SD = 36 ms). The relatively large ROI size was 150 pixels with approximately 60 statistically independent pixels (40%) improving the SD in the ROI by sqrt(60) to approx. 5 ms in the ROI.

The heterogeneity of tissue ranges from focal to globally diffuse disease and associated T1 abnormality. Given adequate precision, the strength of pixel-wise mapping of T1 is the ability to detect small abnormalities and discriminate spatial structures.

## Other factors

### Artifacts

In addition to the factors that influence accuracy and precision, a key limitation is the spatial resolution and the associated partial volume effects, particularly at myocardium-blood and myocardium-fat boundaries. The partial volume effect is dependent on the slice thickness as well as the in-plane resolution. Improved in-plane resolution and decreased slice thickness may be obtained at a sacrifice of SNR. The optimal trade-off has not been determined. Loss of resolution due to cardiac motion blur may result from longer imaging segments and/or imaging during periods of motion. To some extent, T1-mapping error maps can serve as a quality control metric to indicate the presence of poor fitting due to motion [41]. By recognizing the error, it may be possible to adjust the timing or protocol for improved temporal resolution to mitigate motion. Similarly, respiratory motion is unavoidable in clinical practice, even with breath-holding. Respiratory motion correction can be used to mitigate errors to some extent, but residual uncorrected respiratory motion is still problematic, particularly if unrecognized [41].

Artifacts are commonplace in CMR however skilled clinicians are often capable of “reading through” these. Artifacts in quantitative parametric maps are less familiar and recognizing these artifacts will require experience. In addition to motion related artifacts, spatial variation in off-resonance due to B0-field inhomogeneity may lead to artifactual appearance of the T1-map. These may be particularly significant if the subject has devices implanted. Field maps may be acquired as a quality metric [42] but this requires additional data acquisition and some technical expertise from the clinician interpreting the study. In an ideal setting, a complete set of T1, T2, B0, B1+, and water/fat separated images would be acquired for comprehensive tissue characterization, but the acquisition time for all these datasets may be prohibitive.

Saturation recovery schemes such as SASHA that acquire a large number of measurements at short measurement times (<RR) are particularly prone to artifacts since these early images generally have lower SNR. For instance, residual parallel imaging artifacts will have a more significant effect on SASHA acquisition than MOLLI. The same can be said for artifacts related to blood flow. Edge artifacts are also more significant when using higher flip angle excitation, which leads to a distortion of the point spread function due to the transient weighting of k-space during the approach to steady state.

### Field strength

T1 relaxation is dependent on the field strength [9] with a significant increase in T1 from 1.5 T to 3 T field strength. Average native T1 values for normal myocardium measured using inversion recovery are reported to be 962 ± 25 ms at 1.5 T [50] and 1315 ± 39 ms at 3 T [9]. While these values depend on the specific protocols, the field dependence is clearly exhibited. Higher field strength (3 T vs 1.5 T) has some pros and cons for quantifying myocardial T1. A disadvantage of the higher field strength is a greater inhomogeneity of both B0 and B1+ fields, which introduce variations in the apparent T1. However, the higher field strength provides an increased SNR, which may be traded off for decreased errors associated with B0 and B1+ variation by decreasing the SSFP excitation flip angle. A flip angle of 20° for MOLLI based protocols is recommended for 3 T, whereas 35° is widely used at 1.5 T. The SASHA method typically uses a FA = 70° at 1.5 T but is limited to 40°-45° at 3 T due to SAR constraints which significantly decreases the SNR. The myocardial T2 at 3 T is decreased relative to 1.5 T, which introduces greater T1 underestimation due to influence of SSFP readout as well as inversion efficiency. Longer duration RF pulses are generally used at 3 T to reduce SAR thereby increasing the echo spacing, which has negative implications for temporal resolution of single shot imaging particularly for subjects with higher heart rates. Despite challenges of higher field strength for quantifying myocardial tissue T1, mapping at 3 T has been demonstrated to differentiate diffuse disease from normal tissue in clinical studies [18].

### Contrast exchange mechanisms

A number of questions remain to be studied in greater detail. It is important to develop a deeper understanding of the multi-compartment exchange between Gd contrast and the various tissue compartments intracellular, interstitium, and vascular, and the magnetization transfer parameters for exchange between the restricted and free pools in myocardial and blood tissue. These exchange mechanisms influence the accuracy of the T1-measurements and the calculation of extra-cellular volume (ECV) fraction using combined measurement of native T1 and T1 with exogenous contrast. The relative value of native T1 and ECV is a question that is still debated. The magnitude of error in ECV measurement due to intercompartmental exchange mechanisms during Gd washout may not be significant in a clinical context [9,15,55,56].

## Summary

A number of factors have been described that influence the accuracy of T1-mapping. If these factors, which may depend on the protocol or scanner adjustments, are not well controlled, then they can contribute to reduced reproducibility. If these factors are well controlled then the absolute accuracy may be less important and the “apparent” measured T1 might serve as a powerful clinical tool despite the fact that the measurement may not be fully understood. The issue of what is being measured and how it is best used to detect disease is a subject of on-going research at many institutions.

It is difficult to distill the myriad of trade-offs to form recommendations since the sensitivities are multidimensional and interdependent. Nevertheless, in the interest of summarizing the current state-of-knowledge of existing protocols, a summary is provided in Table 4, which at a top-level compares the protocol from a stand-point of accuracy, precision, reproducibility, and artifacts. It is our current opinion that while absolute accuracy is important, that reproducibility and robustness are critical and therefore favor inversion recovery methods at this date. Inversion recovery methods such as MOLLI are in widespread use and are more mature than the saturation recovery counterparts such as SASHA. As saturation recovery methods are studied and possibly optimized further, then it is certainly very attractive to have the potential benefit of improved absolute accuracy.

**Table 4:** Summary of pros and cons of various reported T1-mapping protocols

	**MOLLI 3(3)3(3)5**	**MOLLI 5s(3s)3s**	**MOLLI 4s(1s)3s(1s)2s**	**ShMOLLI**	**SASHA 2p-fit**	**SASHA 3p-fit**
Short breath-hold	-	+	+	+	+	+
HR insensitivity	-	+	+	+	+	+
Absolute accuracy	-	-	-	-	+	++
Precision	++	++	++	+	+	-
Few image artifacts	+	++	++	++	-	-
Reproducibility	-	++	++	++	-	-

(^++^denotes good, + denotes fair, - denotes poor).

## Conclusions

A number of recent studies have shown the sensitivity of T1- and ECV-mapping for detection of disease with diffuse processes involving edema and or fibrosis affecting the interstitium. Many of these studies are population based studies which have demonstrated a correlation between small changes in T1 or ECV with disease or outcomes. T1 and ECV measures have been shown to have important prognostic significance. Quantification has the potential for an objective measurement to detect changes in disease over time or in response to therapy. Translating these exciting results to the reliable diagnosis of individuals where it may impact patient management still has technical challenges. In order to base diagnostic assessments on subtle changes in parameters, the demand for improved reproducibility and measures of confidence are much greater than for population based studies.

Inversion recovery methods such as MOLLI have excellent precision and are highly reproducible when using tightly controlled protocols. The MOLLI method is widely available and is relatively mature. The accuracy of inversion recovery techniques is affected significantly by MT. Despite this, the estimate of apparent inversion recovery time is a sensitive measure, which has been demonstrated to be a useful tool in characterizing tissue and discriminating disease. Saturation recovery methods have the potential to provide a more accurate measurement of T1 that is less sensitive to MT as well as other factors. Saturation recovery techniques are noisier and somewhat more artifact prone and have not demonstrated the same level of reproducibility at this point in time.

A key limitation of T1-mapping for clinical application is the error due to partial volume contamination from blood, which is significant for thin walled structures. Caution must be exercised to ensure adequate spatial resolution is obtained and to recognize less familiar artifacts in parametric maps.

## Competing interests

The authors declare that they have no competing interest.

## Authors’ contributions

PK performed simulations and data analysis, and drafted the manuscript. MSH contributed to data analysis and interpretation of results. All authors participated in revising the manuscript and read and approved the final manuscript.
